# Bushen-Yizhi Formula Alleviates Neuroinflammation via Inhibiting NLRP3 Inflammasome Activation in a Mouse Model of Parkinson's Disease

**DOI:** 10.1155/2018/3571604

**Published:** 2018-08-26

**Authors:** Yousheng Mo, Erjin Xu, Renrong Wei, Baoluu Le, Lei Song, Dongli Li, Yonggen Chen, Xiaotian Ji, Shuhuan Fang, Jiangang Shen, Cong Yang, Qi Wang

**Affiliations:** ^1^Institute of Clinical Pharmacology, Guangzhou University of Chinese Medicine, 12 Airport Road, Baiyun District, Guangzhou 510405, China; ^2^Faculty of Traditional Medicine, University of Medicine and Pharmacy at Ho Chi Minh City, Ho Chi Minh City, Vietnam; ^3^School of Chinese Medicine, The University of Hong Kong, 10 Sassoon Road, Pokfulam, Hong Kong

## Abstract

Parkinson's disease (PD), the second most common neurodegenerative disease, is characterized by the progressive loss of dopaminergic neurons in the substantia nigra. Although the molecular mechanisms underlying dopaminergic neuronal degeneration in PD remain unclear, neuroinflammation is considered as the vital mediator in the pathogenesis and progression of PD. Bushen-Yizhi Formula (BSYZ), a traditional Chinese medicine, has been demonstrated to exert antineuroinflammation in our previous studies. However, it remains unclear whether BSYZ is effective for PD. Here, we sought to assess the neuroprotective effects and explore the underlying mechanisms of BSYZ in a 1-methyl-4-phenyl-1, 2, 3, 6-tetrahydropyridine- (MPTP-) induced mouse model of PD. Our results indicate that BSYZ significantly alleviates the motor impairments and dopaminergic neuron degeneration of MPTP-treated mice. Furthermore, BSYZ remarkably attenuates microglia activation, inhibits NLPR3 activation, and decreases the levels of inflammatory cytokines in MPTP-induced mouse brain. Also, BSYZ inhibits NLRP3 activation and interleukin-1*β* production of the 1-methyl-4-phenyl-pyridinium (MPP^+^) stimulated BV-2 microglia cells. Taken together, our results indicate that BSYZ alleviates MPTP-induced neuroinflammation probably via inhibiting NLRP3 inflammasome activation in microglia. Collectively, BSYZ may be a potential therapeutic agent for PD and the related neurodegeneration diseases.

## 1. Introduction

Parkinson's disease (PD), the second most common age-related neurodegenerative disease after Alzheimer's disease, is affecting approximately 1% of the population over 60 years of age. PD is characterized with motor symptoms such as akinesia, bradykinesia, rigidity, tremor, and the progressive loss of dopaminergic neurons in the substantia nigra and axonal terminals in the striatum [1–3]. PD is reported to be caused by mitochondrial dysfunction, oxidative stress, and chronic inflammation, but its underlying mechanisms are still unknown [4–6].

Increasing reports showed neuroinflammation plays a dominant role in the pathogenesis of PD [6, 7]. It is well known that the activated microglial cells, the major source of proinflammatory factors and cytokines, are closely related to the dopaminergic neurons loss and survival in PD [4, 8]. And the nucleotide binding and oligomerization domain-like (Nod) receptor family pyrin domain-containing 3 (NLRP3) inflammasome, a pathogen recognition receptor (PRR), is highly expressed in microglia, which can be activated by lots of invading pathogens and endogenous danger molecules such as extracellular adenosine 5′-triphosphate (ATP), uric acid crystals, and amyloid-*β* [9–12]. NLRP3 inflammasome is composed of nod-like receptor protein NLRP3, adaptor protein ASC, and pro-caspase-1 [13]. Once activated, it will lead to the autocatalytic cleavage of caspase-1 and ultimately promotes the maturation and release of IL-1*β*. Also, the activation of NLRP3 inflammasome plays a key role in the microglia-mediated neuroinflammation and dopaminergic neuronal degeneration [14]. So, the inhibition of NLRP3 inflammasome activation might be the effective way to alleviate the neuroinflammatory procession in PD [12, 15, 16].

Currently, the first-line drugs such as levodopa can relieve symptoms for Parkinson's disease, but their uses are limited due to the low efficacy and long-term usage side effects [17, 18]. Nowadays, remedies including the natural and herbal medicines with more safety and efficacy are becoming popular for PD [4, 19–22]. BSYZ, a traditional Chinese medicine, is composed of common Cnidium fruit, tree peony bark, ginseng root, Radix Polygoni Multiflori Preparata, Barbary wolfberry fruit, and Fructus Ligustri Lucidi. Our previous studies have confirmed that BSYZ has extensive neuroprotective effects such as antisenesce, antiapoptosis, and alleviation of oxidative stress in various Alzheimer's disease (AD) animal models [23–26]. Our recent investigation further demonstrated BSYZ is effective in reducing age-related neurodegenerative disorders via antineuroinflammation [27]. Therefore, we hypothesize BSYZ may also be effective in PD therapy. So, in this study, we assess BSYZ efficacy in PD with a MPTP-induced mouse model and explore its potential mechanisms in MPP^+^-stimulated BV2 microglia cells.

## 2. Materials and Methods

### 2.1. Preparation of BSYZ

The traditional Chinese medicines, Cnidium monnieri, Panax ginseng, Polygonum multiflorum Thuna, Paeonia suffruticosa Andr, Ligustrum lucidum Ait, and Lycium barbarum, were purchased from Guangxi Yifang Chinese Herbal Medicine Department and identified by Professor Chen Jiannan, a pharmacognosist in the School of Chinese Materia Medica, Guangzhou University of Chinese Medicine. The voucher specimen was deposited at the Institute of Clinical Pharmacology, Guangzhou University of Chinese Medicine with the registration number 20121209.

The BSYZ formula, consisting of six herbs (Cnidium monnieri, Panax ginseng, Polygonum multiflorum Thuna, Paeonia suffruticosa Andr, Ligustrum lucidum Ait, and Lycium barbarum), is mentioned in the ratio of 3 : 3 : 2 : 2 : 2 : 2. Detailed information was presented in Table 1. The extraction process and the qualitatively analysis of BSYZ formula were performed in accordance with our previous study [25].

### 2.2. Animals

Adult male C57BL/6 mice (10-12 weeks, weight 25-30 g) were obtained from Experimental Animal Center of the Guangzhou University of Chinese Medicine (Guangzhou, China). Animals were group-housed under a 12h light/dark cycle with free access to water and food. All animal care and experimentation were approved by the principles and guidelines of the National Institutes of Health Guide for the Care and Use of Laboratory Animals.

### 2.3. BSYZ Treatment

Mice were randomly divided into six groups (N=12 for each group): (1) Control group; (2) MPTP group; (3) MPTP+low-dose BSYZ group, treated with BSYZ 1.46 g/kg; (4) MPTP+middle-dose BSYZ group, treated with BSYZ 2.92 g/kg; (5) MPTP+high-dose BSYZ group, treated with BSYZ 5.84 g/kg; (6) MPTP+ Piroxicam group, treated with Piroxicam 1.25 mg/kg. Mice were pretreated twice daily with BSYZ or Piroxicam through oral gavage as described above for 7 days before MPTP injection, and animals in the control group were treated with 0.9% saline. Mice had intraperitoneal injection with MPTP (18 mg/kg) four times at 2 h intervals to establish PD acute models [4]. Animals in the control group were injected with an equivalent volume of 0.9% saline. After MPTP administration, mice were treated with BSYZ or Piroxicam (Sigma-Aldrich) for 7 consecutive days, accompanied with the behavioral test.

### 2.4. Behavioral Test

The Rotarod test for the motor coordination and balance was conducted using a slight modification [12]. The mice were placed on the rotating rod for 2 min at the speed of 20 rounds per minute. Duration of the mice on the rod (latency to fall) was recorded for further analysis.

For Y-maze test, the total distance in movement and total entries into the arms were evaluated for the autonomous activity, while the spontaneous alternation is used to assess the spatial reference memory. The Y-maze apparatus has three identical arms, and each arm was 35 cm in length, 5 cm in width, and 15 cm in height. The procedures were similar to those described previously [4]. Briefly, each mouse, naive to the maze, was placed at the end of one arm and allowed to move freely through the maze during a 5 min session. Mouse's travel pathway and the total distance of movement were recorded by software (SuperMaze+, Shanghai Xinruan Information Technology Co., Ltd.). The total times of arm entries were recorded manually.

### 2.5. Cell Culture and Drug Treatment

BV-2 microglia cells were obtained from the Chinese Academia Sinica (Shanghai, China). The cells were cultured in DMEM supplemented with 10% fetal bovine serum and antibiotics at 37°C in a humidified incubator supplied with 5% CO_2_. Microglia was activated by MPP^+^* in vitro* according to a previously described method [28]. Briefly, the cells were seeded (1×10^6^cells/well) in a 6-well plate, incubated for 24 h, and treated with MPP^+^ (100 *μ*M) with or without BSYZ (50 *μ*g/mL, 100 *μ*g/mL or 200 *μ*g/mL) for another 6 h. At the same time, a BSYZ (200 *μ*g/ml) group was set as the normal control group and MCC950 (MCE), a selective NLRP3 inhibitor, was set as the positive control. After that, the cells were stimulated with 2mM ATP (Sigma-Aldrich) for 45 min.

### 2.6. Western Blot Analysis

Western blot assays for the target proteins were performed by using the common approach. For cells assays, the cells were washed twice with PBS, placed at 4°C, and lysed for 10 min in sodium dodecyl sulfate (SDS) lysis buffer (containing protease inhibitor cocktail and phosphatase inhibitor cocktail). For animal assays, the midbrain was rapidly removed, washed with cold PBS (10 mmol·L^−1^, pH 7.4), and lysed with SDS buffer for 0.5 h on ice. The lysates were centrifuged at 12000 rpm for 15 min at 4°C and the supernatants were collected for further analysis. Protein concentrations were determined by using BCA Protein Assay Kit (Millipore, American). Equal amounts of protein (20 *μ*g or 40 *μ*g) were separated by 10% SDS PAGE, and the resolved protein was transferred to polyvinylidene difluoride membranes (Millipore, American). The membranes were soaked with 7% (w/v) skim milk for 1.5 h at room temperature, incubated at 4°C overnight with primary antibodies (rabbit anti-NLRP3, mouse anti-ASC, rabbit anti-caspase-1, mouse anti IL-1*β* (1:1000, Celling Signaling Technology), and mouse anti-*β*-actin (1:20000, sigma)). The membranes were washed 3 times with TBST with Tween 20 for 10 min each time, followed by incubation with the peroxidase-conjugated anti-mouse (1:4000) or anti-rabbit IgG (1:4000) for 1.5 h at room temperature. The blots were visualized by using an ECL Western blot detection kit (Millipore, WBKLS0500). Image J (National Institutes of Health, Bethesda, Maryland, USA) was used to evaluate the densitometry.

### 2.7. Immunofluorescence

After perfusion with 4% paraformaldehyde, brains were removed, postfixed in the same solution at 4°C overnight, cryoprotected in 30% sucrose, and finally 30 *μ*m coronal sections were obtained according to a previous report [29]. In immunofluorescence detection, antigen retrieval was performed by 70°C heating of the sections in sodium citrate buffer (10 mM trisodium citrate, 0.5% Tween-20 in H_2_O, pH 6.0) for 30 min. The sections were blocked with 10% goat serum (with 0.5% Triton X-100) in Tris-buffered saline for 20 min and labeled with TH (Abcam, 1:2000), GFAP antibody (Abcam, 1:200), Iba-1 antibody (Woko, 1:200), or CD68 (Abcam, 1:200), in blocking buffer for 48 h at 4°C. After that, the slides were washed with PBS for three times (5 min each time), incubated with anti-rabbit secondary antibodies conjugated to Alexa Fluor 594 (Cell Signaling Technology, 1:1000) or Alexa Fluor 488 (Cell Signaling Technology, 1:1000) for 1 h. Thereafter, the slides were washed with PBS for three times again, and the sections were stained with DAPI. Finally, the sections were covered with coverslips and mounted with anti-fade fluorescence mounting medium (Beyotime Biotechnology) and observed under a fluorescence microscope (Model DMi8, Leica, Germany).

### 2.8. Nissl Staining

Nissl staining was used to determine the density of dopaminergic neurons cells in SNpc. Briefly, Sections were incubated with cresyl violet solution for 20 minutes. After natural drying, the sections were mounted with neutral balsam and observed under the microscope.

### 2.9. Cells Counting

The total numbers of TH-positive cells, Iba-1/CD68 labeled microglia, and GFAP-positive cells in the entire extent of SNpc were counted according to a previous report [29]. Briefly, each brain contained 6 serial sections at 6 intervals and 4 mouse brains per group. One series of sections per mouse was selected for immunohistochemical staining. The stereological analyses were using the Optical Fractionator method with Microbrightfield Stereo-Investigator software (Stereo-Investigator software, Microbrightfield, VT, USA). All stereological analyses were performed under the ×200 magnification.

### 2.10. Real-Time PCR

Total RNA was isolated from midbrain by using RNAiso Plus (Takara) following the standard protocol. For quality control, RNA purity was quantified by the NanoDrop 2000 spectrophotometer (Thermo Scientific). Total RNA (1 *μ*g) was reverse-transcribed to cDNA by using a Reverse Transcription Kit (Takara). Real-time quantitative PCR was performed with SYBR® Premix Ex TaqTM II (Takara) and the CFX96TM Real-Time PCR Detection System (Bio-Rad). The following primers were designed and synthesized by Life Technologies: IL-1*β*, forward, GAA ATG CCA CCT TTT GAC AGT G, and reverse TGG ATG CTC TCA TCA GGA CAG; IL6, forward TAG TCC TTC CTA CCC CAA TTT CC, and reverse TTG GTC CTT AGC CAC TCC TTC; TNF*α*, forward, CAG GCG GTG CCT ATG TCT C, and reverse CGA TCA CCC CGA AGT TCA GTA G; Actb, forward, GGC TGT ATT CCC CTC CAT CG, and reverse CCA GTT GGT AAC AAT GCC ATG T. GAPDH, forward, AGG TCG GTG TGA ACG GAT TTG, and reverse TGT AGA CCA TGT AGT TGA GGT CA.

All samples were analyzed and normalized with the expression levels of two housekeeping genes (*β*-actin and GAPDH). The mRNA levels were analyzed and quantified with the 2−ΔΔCt method by CFX Manager Software provided by the CFX96TM Real-Time PCR Detection System (BioRad Laboratories, Inc.).

### 2.11. Statistical Analysis

All data were expressed as mean ± standard error. Statistical results were obtained using the statistical software SPSS 17.0. One-way analysis of variance (ANOVA) was used to analyze statistical differences between groups or Student's* t*-test was performed as appropriate.* P*<0.05 was considered significantly different.

## 3. Results

### 3.1. BSYZ Alleviates Behavioral Impairment in MPTP-Induced PD Mice

**Figure 1:**
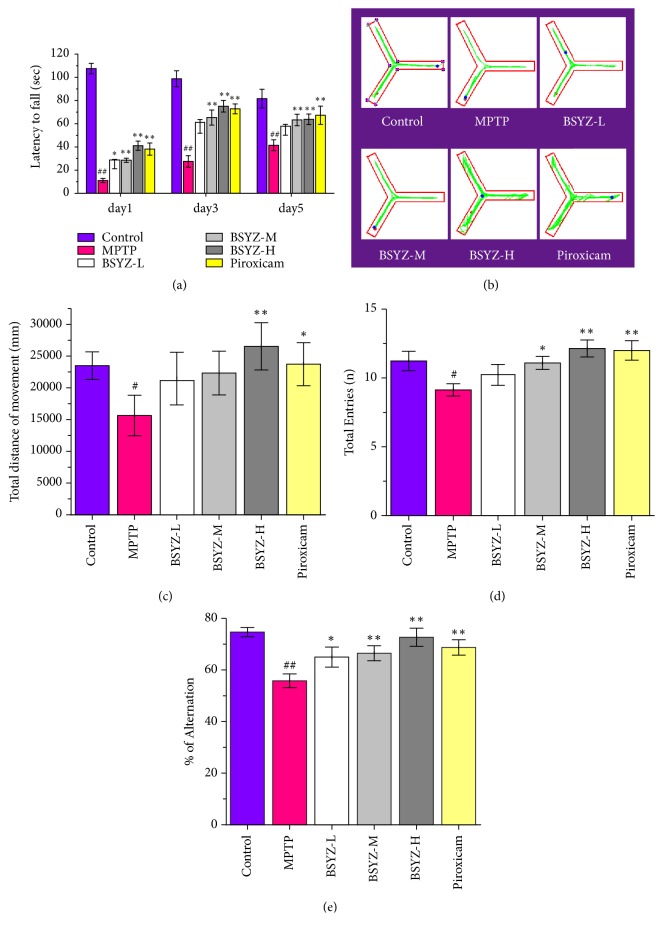
**BSYZ improves the motor impairments in MPTP-induced mice.** (a) Latency to fall in Rotarod test performed on days 1, 3, and 5 after MPTP injection. (b) Mouse's travel pathway, (c) the total distance of movement, (d) total entries, and (e) percentage alternations in Y-maze were performed on Day 7 after MPTP injection. Values are mean ± standard error (n = 12 per group). Experimental values were expressed as means ± SEM, ^#^*P* < 0.05 and ^##^*P* < 0.01 versus control group; *∗P* < 0.05 and *∗∗P* < 0.01 versus MPTP-treated group.

Rotarod test and Y-maze test are often used to evaluate the motor deficiency. As shown in Figure 1(a), mice with MPTP injections exhibited a dramatically reduced latency relative to that of control. However, mice pretreated with BSYZ (middle dose, 2.92 g/kg or high dose, 5.84 g/kg) displayed an increase latency.

In this study, we found that mice in MPTP group showed a remarkable decline in total distance of movement, total entries relative to those of the control group in Y-maze test (Figures 1(c)–1(e)). However, MPTP mice with BSYZ in both high dose and middle dose could alleviate this decrease. These results confirmed that BSYZ administration could alleviate MPTP-induced motor impairment. Interestingly, BSYZ also increased the percentage of alternation in Y-maze at the same time.

### 3.2. BSYZ Protects Dopaminergic Neurons against MPTP-Induced Neurodegeneration

**Figure 2:**
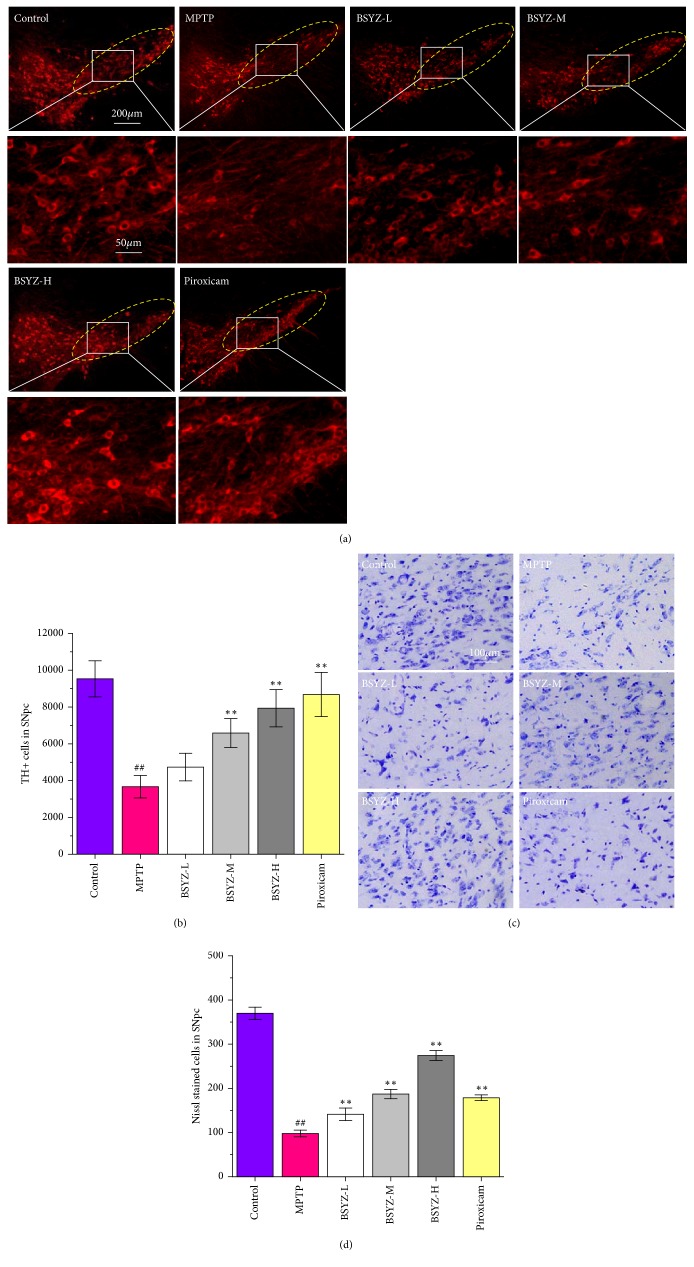
**BSYZ protects against tyrosine hydroxylase (TH) depletion and dopaminergic neurons neurodegeneration in MPTP-induced mice.** (a) Representative images of TH-positive cell immunoreactivity in substantia nigra pars compacta (SNpc) sections. (b) Statistical results for the number of TH-positive neurons in the SNpc. (c) Nissl^+^ neurons in SNpc area and (d) statistical results were shown by Nissl Staining (n = 4 per group). Scale bar: 100 *μ*m. Experimental values were expressed as means ± SEM, ^#^*P* < 0.05 and ^##^*P* < 0.01 versus Control group; *∗P* < 0.05 and *∗∗P* < 0.01 versus MPTP-treated group.

Next, we detected tyrosine hydroxylase (TH) in SNpc by immunofluorescence, with the purpose to confirm whether BSYZ protects dopaminergic neurons from MPTP damage. The administration of MPTP resulted in an obviously loss of TH-positive neurons in SNpc, while BSYZ treatment significantly increased the number of TH-positive neurons in a dose-dependent manner (Figures 2(a) and 2(b)). Correspondingly, the Nissl positive neurons in MPTP group were reduced remarkably. But BSYZ treatment (1.46, 2.92, or 5.84 g/kg) significantly restored the Nissl positive neurons (Figures 2(c) and 2(d)). Taken together, these results indicate that BSYZ exerts a beneficial effect on dopaminergic neuronal degeneration. Similarly, the treatment with Piroxicam (1.25 mg/kg) was also able to alleviate the dopaminergic neurons neurodegeneration.

### 3.3. BSYZ Attenuates Neuroinflammation in the MPTP-Induced PD Mice

**Figure 3:**
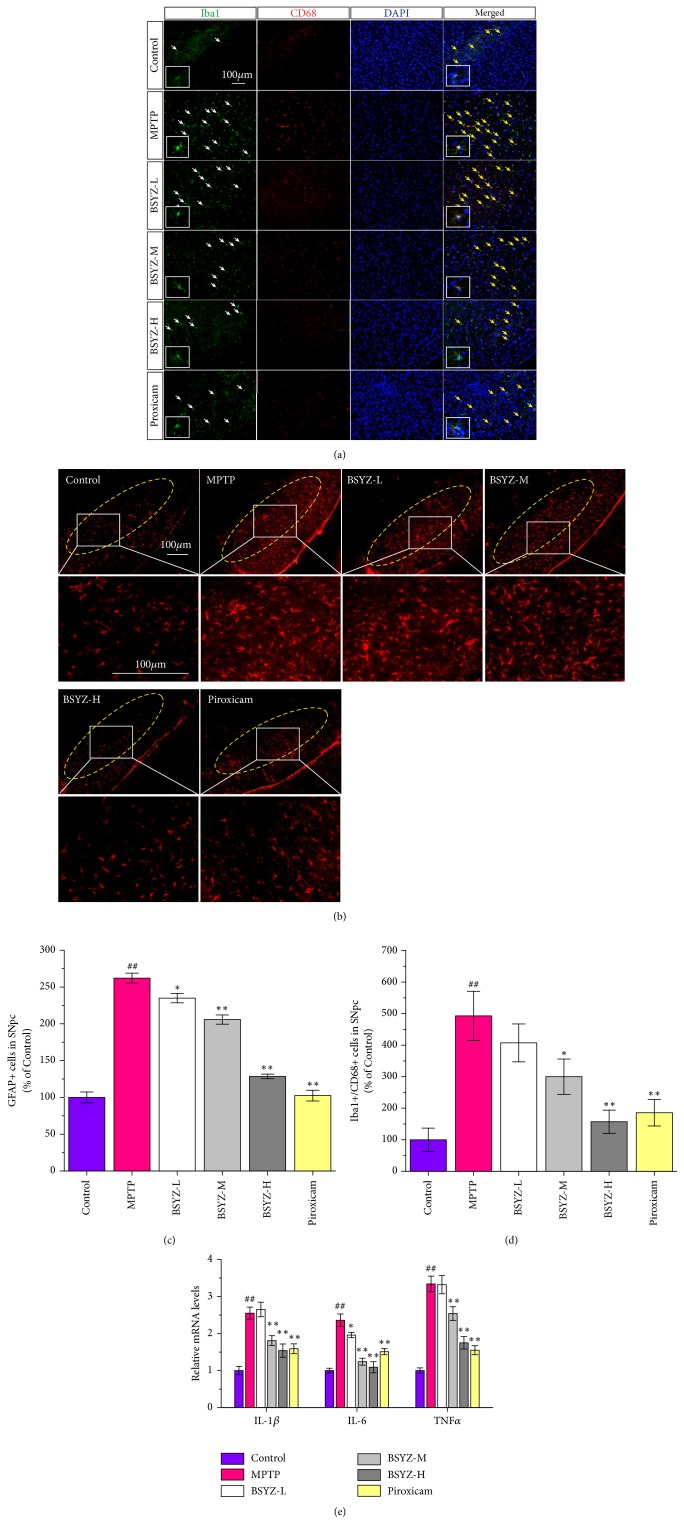
**BSYZ attenuates neuroinflammation in the MPTP-induced mice.** (a) Representative immunofluorescence images of the SNpc stained with anti-Iba-1 (green) and anti-CD68 antibody (red). (b) Representative immunofluorescence images of the SNpc section stained with anti-GFAP antibody. (c) Statistical results for the relative GFAP-positive cells in the SNpc. (d) Statistical results for the relative number of Iba-1 and CD68 double positive cells in the SNpc. (e) The mRNA levels of proinflammatory factors in midbrain. Experimental values were expressed as means ± SEM, ^#^*P* < 0.05, ^##^*P* < 0.01 versus control group, *∗P* < 0.05, *∗∗P* < 0.01 versus MPTP group.

Microglia and astrocyte are the main players in the neuroinflammatory process in the neurodegenerative diseases [13]. Firstly, we used the microglia marker Iba-1 and CD68 to label microglia and the astrocyte marker glial fibrillary acidic protein (GFAP) to label astrocyte in SNpc. As shown in Figure 3(a), more microglia cells labeled with Iba1 were found with large somas and numerous short branches (white arrows shown in Figure 3(a)) in the SNpc confirming the activation of microglia after MPTP injections. BSYZ administration remarkably inhibited the microglia activation as evidenced by the decreased number of Iba-1 and CD68 double positive cells (yellow arrows shown in Figure 3(d)). In addition, MPTP also induced an increase of GFAP-positive cells in the SNpc area, an indicator of the proliferation and activation of astrocytes, but this can be alleviated by BSYZ treatment (Figures 3(b) and 3(c)). These results suggest that BSYZ treatment could inhibit the microglia activation and astrocyte proliferation in SNpc of MPTP mice.

Neuroinflammation is also characterized by increasing production of proinflammatory factors in the brain [4, 12]. So, the levels of inflammatory factors such as IL-1*β*, IL-6, and TNF*α* in the midbrain region were detected by qPCR. The results indicated that MPTP treatment increased the mRNA levels of proinflammatory cytokines including IL-1*β*, IL-6, and TNF*α*. BSYZ attenuated the upregulation of IL-1*β*, IL-6, and TNF*α* in a dose-dependent manner (Figure 3(e)). Similarly, the treatment with Piroxicam was also able to alleviate the glial activation and decrease the level of proinflammatory cytokines in the MPTP mice. These results suggest that BSYZ can inhibit the neuroinflammation in the MPTP mice.

### 3.4. BSYZ Suppresses NLRP3 Inflammasome Activation in the MPTP-Induced PD Mice

**Figure 4:**
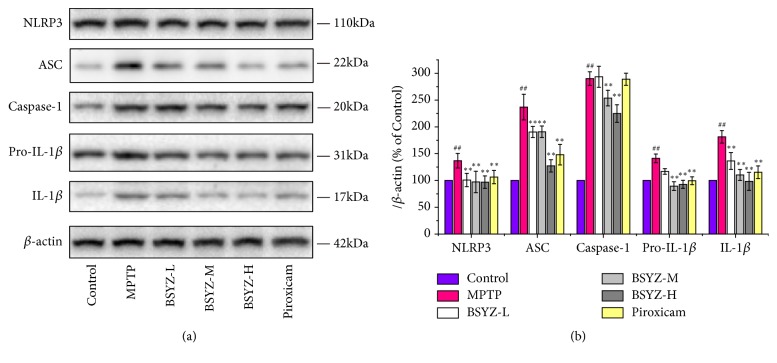
**BSYZ suppresses NLRP3 inflammasome signaling in MPTP-induced mice.** (a) NLRP3 inflammasomes including NLRP3, ASC, caspase-1, pro-IL-1*β*, and IL-1*β* in midbrain were measured by Western blotting and *β*-actin was detected as an internal control. (b) Quantification data of NLRP3 inflammasome level were detected by Western blot analysis (n = 3 per group). Experimental values were expressed as means ± SEM, ^#^*P* < 0.05, ^##^*P* < 0.01 versus control group. *∗P* < 0.05, *∗∗P* < 0.01 versus MPTP group.

The NLRP3 inflammasome signaling pathway is a major contributor to the neuroinflammatory process in the nervous system disease [30]. The NLRP3 inflammasome level in MPTP-induced mice model was detected to explore BSYZ efficacy to inhibit neuroinflammation. Western blotting analysis revealed that NLRP3 inflammasome was activated in the midbrain of MPTP mice with more expressions of NLRP3, ASC, caspase-1, pro-IL-1*β*, and IL-1*β*. However, this phenomenon was reversed for the mice with BSYZ treatment (Figure 4), confirming that BSYZ can inhibit the NLRP3 inflammasome activation in PD mice model.

### 3.5. BSYZ Suppresses NLRP3 Inflammasome Activation in MPP^+^-Stimulated BV2 Cells

**Figure 5:**
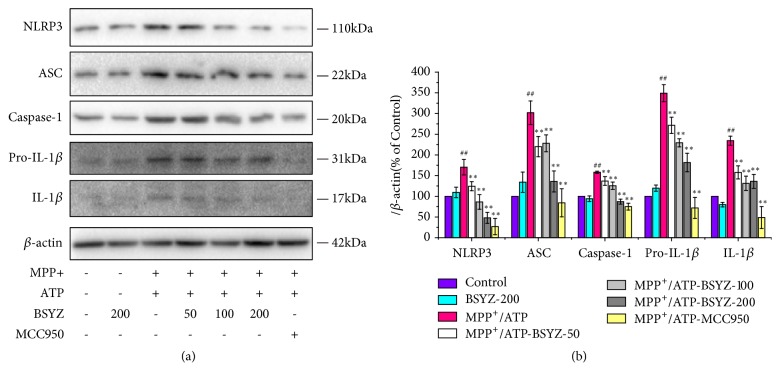
**BSYZ suppresses NLRP3 inflammasome signaling in MPP** ^**+**^ **-treated BV2 cells.** (a) NLPR3 inflammasome including NLPR3, ASC, caspase-1, pro-IL-1*β*, and IL-1*β* were detected by Western blotting and *β*-actin was detected as an internal control (n =3 per group). (b) Statistical results of NLRP3 inflammasome level were detected by Western blot analysis (n = 3 per group). Experimental values were expressed as means ± SEM. ^#^*P* < 0.05, ^##^*P* < 0.01 versus control group. *∗P* < 0.05, *∗∗P* < 0.01 versus MPTP group.

Previous studies showed that NLRP3 inflammasome is highly expressed in microglia. So, BV2 cells, an immortalized murine microglia cell line, were selected to assess NLRP3 inflammasome role in MPP^+^-stimulated microglia activation. Western blot results showed that NLRP3 inflammasome was activated after MPP^+^ and ATP stimulation. But NLRP3 inflammasome component, including NLRP3, caspase-1, ASC, pro-IL-1*β*, and IL-1*β*, were downregulated by BSYZ in a concentration-dependent manner (Figure 5). The positive group with the treatment of MCC-950 (100 nM), a NLRP3 inflammasome inhibitor, also inhibited the NLRP3 inflammasome activation, while the expression of NLRP3 inflammasome was not obviously affected by BSYZ alone. These data further confirm that BSYZ indeed can inhibit NLRP3 inflammasome activation in microglia.

## 4. Discussion

Aging is the main cause to lead the occurrence of AD and PD. BSYZ is usually used in the treatment of dementia and aging-related memory deficiency in China [23]. However, it remains unknown whether BSYZ is also effective for PD. In this study, we used a classic systemic PD model based on the administration of MPTP to explore the effect of BSYZ. Our results indicate that BSYZ is neuroprotective to against the MPTP-induced motor deficiency and dopaminergic neuronal degeneration. Moreover, BSYZ can alleviate MPTP-induced neuroinflammation via inhibiting the NLRP3 inflammasome activation in substantia nigra. In addition,* in vitro* study further reveals that BSYZ inhibits the NLRP3 inflammasome activation in MPP^+^-stimulated BV2 microglia.

In the development of PD drugs, MPTP mouse model is often used to mimic the pathological and behavioral changes of PD patients [31]. In this study, we observed that the mice in MPTP-treated group displayed a decreased latency to fall in Rotarod experiment. Y-maze test has been previously utilized to assess locomotor activity and recognition memory [32, 33]. Neurotoxins, including MPTP, 6-hydroxydopamine and rotenone, not only induce motor impairment, but also affect cognitive networks including the learning and memory functions [34–36]. In this study, we found that the mice in MPTP-induced group exhibited a remarkable motor impairment and memory deficits as evidenced by both the decrease of total distance of movement, total arms entries, and the percentage of alternation in Y-maze test. BSYZ administrations alleviated MPTP-induced motor impairment. Interestingly, BSYZ also increased the percentage of alternation in Y-maze at the same time. That might be associated with the treatment effects of BSYZ on learn and memory deficits related diseases [23, 24, 26]. Consistent with these data, the mice with MPTP treatment also exhibited obviously dopaminergic neuronal degeneration with decreased TH-positive cells and Nissl positive staining neurons in SNpc area. Interestingly, BSYZ significantly improves the dopaminergic neurons survival condition of MPTP-induced mice. These results indicate that BSYZ exerts a beneficial effect against the motor deficits and dopaminergic neuronal degeneration.

Microglia activation plays a key role in the PD neuroinflammation process [4, 8]. Increasing evidence showed that loss of dopaminergic neurons in the substantia nigra relates to the activated microglia in both PD patients and animal models [37–39]. Activated microglia will release large amounts of inflammatory cytokines such as IL-1*β*, TNF-*α* [40–44], which are detrimental to the survival of DA neurons. Therefore, inhibition of inflammatory response originated from activated microglia may be a promising strategy to protect dopaminergic neurons from inflammatory injury in PD. Recently, astrocyte proliferation was also confirmed to be concomitant with neuronal death in PD animal models [13]. In this study, a large number of activated microglia cells were observed with increased levels of proinflammatory cytokines such as TNF-*α*, IL-6, and IL-1*β* in the MPTP-induced mouse brains. Also, astrocyte proliferations, another inflammatory signature, were observed by GFAP immunohistochemistry. However, BSYZ treatment remarkably suppressed the activation of microglia and astrocyte proliferations in the brains of PD animal. Furthermore, BSYZ effectively reduced the mRNA levels of inflammatory factors. These results confirm BSYZ is effective in suppressing the neuroinflammatory responses induced by MPTP.

Recently, NLRP3 inflammasome was reported to play a key role in the microglia-mediated neuroinflammation and dopaminergic neuronal degeneration of PD [12, 45]. More and more evidences have indicated that NLRP3 inflammasomes were assembled and activated in both PD patients and PD animals with elevated NLRP3, caspase-1, and IL-1*β* level in the serum and brain tissues [14, 30, 46]. Therefore, inhibiting NLRP3 inflammasome activation might be the effective strategy to slow down the dopaminergic neuron degeneration in PD. In this study, we found that MPTP induces high level of NLRP3, IL-1*β* production, and capase-1 cleavage in midbrain, confirming NLRP3 inflammasome was activated after MPTP injection [12]. In order to further investigate BSYZ effect on microglia NLRP3 inflammasome, we stimulated the NLRP3 inflammasome in BV-2 microglia cells by MPP^+^* in vitro*. We found the NLRP3 inflammasomes were also activated with increased level of NLRP3, caspase-1, ASC, pro- IL-1*β*, and IL-1*β*. What is interesting, BSYZ downregulated the expression of NLRP3, caspase-1, ASC, pro- IL-1*β*, and IL-1*β* both in MPTP-induced mice and MPP^+^ stimulated microglia* in vitro*, confirming BSYZ is effective in inhibiting the NLRP3 activation in PD models both* in vivo* and* in vitro* and BSYZ may protect against dopaminergic neuronal injuries via deactivation of microglia NLRP3 inflammasome.

**Figure 6:**
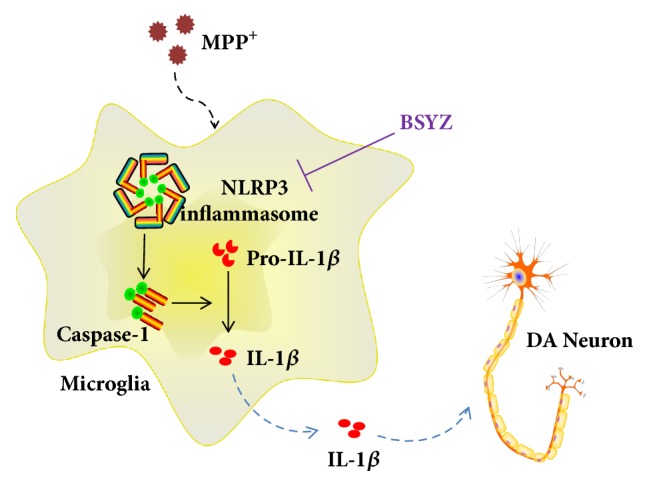
Mechanisms that BSYZ protects dopaminergic neurons from inflammation via inhibiting NLRP3 inflammasome in microglia.

NLRP3 inflammasome-mediated IL-1*β* production requires two signals [12]. The first signal induces nuclear transcription factor-*κ*B (NF-*κ*B) to increase the expression of NLRP3 and pro-IL-1*β*, which is a prerequisite for inflammasome activation. The second signal directly activates the NLRP3 inflammasome to induce caspase-1 cleavage, leading to the maturation of IL-1*β*. Therefore, decreasing the translocation of NF-*κ*B or inhibiting the expression of NLRP3 is effective in suppressing NLRP3 inflammasome activation. In this study, we found that BSYZ decreases the expression of NLRP3 in MPTP-induced mice model and MPP^+^ induced BV2 microglia (Figure 6). Thus, BSYZ may inhibit the NLRP3 inflammasome activation may via suppressing NLRP3 levels directly. In addition, NLRP3 inflammasome can be also activated by ROS which is can be produced by oxidative stress [47, 48]. Our previous studies have shown that BSYZ is effective against oxidative damage [24], so it is possible that BSYZ suppresses NLRP3 inflammasome activation via attenuating the oxidative stress. Nevertheless, the mechanisms by which BSYZ suppress NLRP3 inflammasome remain to be further elucidated.

## 5. Conclusions

In summary, our findings reveal that BSYZ protects against the motor deficits, dopaminergic neuronal degeneration, and neuroinflammation possibly through inhibiting NLRP3 inflammasome activation in microglia. BSYZ may be a promising medicine for NLRP3 inflammasome-driven inflammatory diseases such as PD.

## Figures and Tables

**Table 1:** Constituents of BSYZ.

Botanical name	English name	Chinese name	Used part	Ratio
Cnidium monnieri L.	common cnidium fruit	She-Chuang- Zi	fruit	3
Panax ginseng C. A. Mey.	ginseng	Ren-Shen	rhizome	3
Polygonum multiflorum Thuna	tuber fleeceflower root	He-Shou-Wu	radix	2
Paeonia suffruticosa Andr	tree peony bark	Mu-Dan-Pi	cortex	2
Ligustrum lucidum Ait	glossy privet fruit	Nv-Zhen-Zi	fruit	2
Lycium barbarum L	Barbary wolfberry fruit	Gou-Qi-Zi	fruit	2

## Data Availability

The data used to support the findings of this study are available from the corresponding author upon request.

## References

[1] Gasser T. (2009). Molecular pathogenesis of Parkinson disease: Insights from genetic studies. *Expert Reviews in Molecular Medicine*.

[2] Schapira A. H. V., Bezard E., Brotchie J. (2006). Novel pharmacological targets for the treatment of Parkinson's disease. *Nature Reviews Drug Discovery*.

[3] Connolly B. S., Lang A. E. (2014). Pharmacological treatment of Parkinson disease: a review. *Journal of the American Medical Association*.

[4] Kim B., Koppula S., Kumar H. (2015). alpha-Asarone attenuates microglia-mediated neuroinflammation by inhibiting NF kappa B activation and mitigates MPTP-induced behavioral deficits in a mouse model of Parkinson's disease. *Neuropharmacology*.

[5] Hirsch E. C., Vyas S., Hunot S. (2012). Neuroinflammation in Parkinson's disease. *Parkinsonism & Related Disorders*.

[6] Hirsch E. C., Hunot S. (2009). Neuroinflammation in Parkinson's disease: a target for neuroprotection?. *The Lancet Neurology*.

[7] Wang Q., Liu Y., Zhou J. (2015). Neuroinflammation in Parkinson's disease and its potential as therapeutic target. *Transl Neurodegener*.

[8] Stephenson J., Nutma E., van der Valk P., Amor S. (2018). Inflammation in CNS neurodegenerative diseases. *The Journal of Immunology*.

[9] Heneka M. T., Kummer M. P., Stutz A. (2013). NLRP3 is activated in Alzheimer's disease and contributes to pathology in APP/PS1 mice. *Nature*.

[10] Shi F., Kouadir M., Yang Y. (2015). NALP3 inflammasome activation in protein misfolding diseases. *Life Sciences*.

[11] Youm Y. H., Nguyen K. Y., Grant R. W. (2015). The ketone metabolite beta-hydroxybutyrate blocks NLRP3 inflammasome-mediated inflammatory disease. *Nature Medicine*.

[12] Fan Z., Liang Z., Yang H., Pan Y., Zheng Y., Wang X. (2017). Tenuigenin protects dopaminergic neurons from inflammation via suppressing NLRP3 inflammasome activation in microglia. *Journal of Neuroinflammation*.

[13] Liu Y., Hu J., Wu J. (2012). *α*7 nicotinic acetylcholine receptor-mediated neuroprotection against dopaminergic neuron loss in an MPTP mouse model via inhibition of astrocyte activation. *Journal of Neuroinflammation*.

[14] Zhou Y., Lu M., Du R. H. (2016). MicroRNA-7 targets Nod-like receptor protein 3 inflammasome to modulate neuroinflammation in the pathogenesis of Parkinson's disease. *Mol Neurodegener*.

[15] Qiao C., Zhang L.-X., Sun X.-Y., Ding J.-H., Lu M., Hu G. (2017). Caspase-1 Deficiency Alleviates Dopaminergic Neuronal Death via Inhibiting Caspase-7/AIF Pathway in MPTP/p Mouse Model of Parkinson’s Disease. *Molecular Neurobiology*.

[16] Lawana V., Singh N., Sarkar S. (2017). Involvement of c-Abl Kinase in Microglial Activation of NLRP3 Inflammasome and Impairment in Autolysosomal System. *Journal of Neuroimmune Pharmacology*.

[17] Stocchi F., Marconi S. (2010). Factors associated with motor fluctuations and dyskinesia in Parkinson disease: Potential role of a new melevodopa plus Carbidopa formulation (Sirio). *Clinical Neuropharmacology*.

[18] Borovac J. A. (2016). Side effects of a dopamine agonist therapy for Parkinson’s disease: A mini-review of clinical pharmacology. *Yale Journal of Biology and Medicine*.

[19] Zhang J., Zhang Z., Bao J. (2017). Jia-Jian-Di-Huang-Yin-Zi decoction reduces apoptosis induced by both mitochondrial and endoplasmic reticulum caspase12 pathways in the mouse model of Parkinson's disease. *Journal of Ethnopharmacology*.

[20] Li H., Park G., Bae N., Kim J., Oh M. S., Yang H. O. (2015). Anti-apoptotic effect of modified Chunsimyeolda-tang, a traditional Korean herbal formula, on MPTP-induced neuronal cell death in a Parkinson's disease mouse model. *Journal of Ethnopharmacology*.

[21] Zhang Y., Gong X.-G., Wang Z.-Z. (2016). Protective effects of DJ-1 medicated Akt phosphorylation on mitochondrial function are promoted by Da-Bu-Yin-Wan in 1-methyl-4-phenylpyridinium-treated human neuroblastoma SH-SY5Y cells. *Journal of Ethnopharmacology*.

[22] Li X., Zhang Y., Wang Y. (2017). The Mechanisms of Traditional Chinese Medicine Underlying the Prevention and Treatment of Parkinson's Disease. *Frontiers in Pharmacology*.

[23] Zhang S.-J., Xu T.-T., Li L. (2017). Bushen-Yizhi formula ameliorates cognitive dysfunction through SIRT1/ER stress pathway in SAMP8 mice. *Oncotarget *.

[24] Hou X.-Q., Wu D.-W., Zhang C.-X. (2014). Bushen-Yizhi formula ameliorates cognition deficits and attenuates oxidative stress-related neuronal apoptosis in scopolamine-induced senescence in mice. *International Journal of Molecular Medicine*.

[25] Hou X.-Q., Zhang L., Yang C. (2015). Alleviating Effects of Bushen-Yizhi Formula on Ibotenic Acid-Induced Cholinergic Impairments in Rat. *Rejuvenation Research*.

[26] Cai H., Luo Y., Yan X. (2018). The Mechanisms of Bushen-Yizhi Formula as a Therapeutic Agent against Alzheimer’s Disease. *Scientific Reports*.

[27] Hou X., Song H., Chen Y. (2018). Effects of Bushen-Yizhi formula on age-related inflammation and oxidative stress in senescence-accelerated mice. *Molecular Medicine Reports*.

[28] Lee E., Hwang I., Park S. (2018). MPTP-driven NLRP3 inflammasome activation in microglia plays a central role in dopaminergic neurodegeneration. *Cell Death & Differentiation*.

[29] Ip C. W., Cheong D., Volkmann J. (2017). Stereological Estimation of Dopaminergic Neuron Number in the Mouse Substantia Nigra Using the Optical Fractionator and Standard Microscopy Equipment. *Journal of Visualized Experiments*.

[30] Sarkar S., Malovic E., Harishchandra D. S. (2017). Mitochondrial impairment in microglia amplifies NLRP3 inflammasome proinflammatory signaling in cell culture and animal models of Parkinson’s disease. *npj Parkinson's Disease*.

[31] Duty S., Jenner P. (2011). Animal models of Parkinson's disease: A source of novel treatments and clues to the cause of the disease. *British Journal of Pharmacology*.

[32] Yoshikawa M., Soeda Y., Michikawa M., Almeida O. F. X., Takashima A. (2018). Tau depletion in APP transgenic mice attenuates task-related hyperactivation of the hippocampus and differentially influences locomotor activity and spatial memory. *Frontiers in Neuroscience*.

[33] Walrave L., Vinken M., Albertini G., de Bundel D., Leybaert L., Smolders I. J. (2016). Inhibition of connexin43 hemichannels impairs spatial short-term memory without affecting spatial working memory. *Frontiers in Cellular Neuroscience*.

[34] Zhao T. T., Kim K. S., Shin K. S. (2017). Gypenosides ameliorate memory deficits in MPTP-lesioned mouse model of Parkinson's disease treated with L-DOPA. *BMC Complementary and Alternative Medicine*.

[35] Zhu G., Li J., He L., Wang X., Hong X. (2015). MPTP-induced changes in hippocampal synaptic plasticity and memory are prevented by memantine through the BDNF-TrkB pathway. *British Journal of Pharmacology*.

[36] More S., Kumar H., Cho D., Yun Y., Choi D. (2016). Toxin-Induced Experimental Models of Learning and Memory Impairment. *International Journal of Molecular Sciences*.

[37] Qian L., Wu H., Chen S. (2011). *β*2-Adrenergic Receptor Activation Prevents Rodent Dopaminergic Neurotoxicity by Inhibiting Microglia via a Novel Signaling Pathway. *The Journal of Immunology*.

[38] Liu J., Huang D., Xu J. (2015). Tiagabine Protects Dopaminergic Neurons against Neurotoxins by Inhibiting Microglial Activation. *Scientific Reports*.

[39] Gu R., Zhang F., Chen G. (2017). Clk1 deficiency promotes neuroinflammation and subsequent dopaminergic cell death through regulation of microglial metabolic reprogramming. *Brain, Behavior, and Immunity*.

[40] Koprich J. B., Reske-Nielsen C., Mithal P., Isacson O. (2008). Neuroinflammation mediated by IL-1*β* increases susceptibility of dopamine neurons to degeneration in an animal model of Parkinson's disease. *Journal of Neuroinflammation*.

[41] Dobbs R. J., Charlett A., Purkiss A. G., Dobbs S. M., Weller C., Peterson D. W. (1999). Association of circulating TNF-*α* and IL-6 with ageing and parkinsonism. *Acta Neurologica Scandinavica*.

[42] Qin L., Liu Y., Hong J., Crews F. T. (2013). NADPH oxidase and aging drive microglial activation, oxidative stress, and dopaminergic neurodegeneration following systemic LPS administration. *Glia*.

[43] Chang R. C. C., Hudson P., Wilson B., Haddon L., Hong J.-S. (2000). Influence of neurons on lipopolysaccharide-stimulated production of nitric oxide and tumor necrosis factor-*α* by cultured glia. *Brain Research*.

[44] Wu D., Teismann P., Tieu K. (2003). NADPH oxidase mediates oxidative stress in the 1-methyl-4-phenyl-1,2,3,6-tetrahydropyridine model of Parkinson's disease. *Proceedings of the National Acadamy of Sciences of the United States of America*.

[45] Lee E., Hwang I., Park S. MPTP-driven NLRP3 inflammasome activation in microglia plays a central role in dopaminergic neurodegeneration. *Cell Death & Differentiation*.

[46] Mao Z., Liu C., Ji S. (2017). The NLRP3 Inflammasome is Involved in the Pathogenesis of Parkinson’s Disease in Rats. *Neurochemical Research*.

[47] Tschopp J., Schroder K. (2010). NLRP3 inflammasome activation: the convergence of multiple signalling pathways on ROS production?. *Nature Reviews Immunology*.

[48] Abais J. M., Xia M., Zhang Y., Boini K. M., Li P.-L. (2015). Redox regulation of NLRP3 inflammasomes: ROS as trigger or effector?. *Antioxidants & Redox Signaling*.

